# The signaling pathway of hypoxia inducible factor in regulating gut homeostasis

**DOI:** 10.3389/fmicb.2023.1289102

**Published:** 2023-10-27

**Authors:** Wei Liu, Xueni Fan, Boshuo Jian, Dongxu Wen, Hongzhuang Wang, Zhenjiang Liu, Bin Li

**Affiliations:** ^1^Institute of Animal Husbandry and Veterinary, Tibet Academy of Agricultural and Animal Husbandry Sciences, Key Laboratory of Animal Genetics and Breeding on Tibetan Plateau, Ministry of Agriculture and Rural Affairs, Lhasa, China; ^2^School of Public Health, Lanzhou University, Lanzhou, China; ^3^National Engineering Laboratory for AIDS Vaccine, School of Life Sciences, Jilin University, Changchun, China

**Keywords:** hypoxia, HIFs, signaling pathways, inflammation, gut homeostasis

## Abstract

Hypoxia represent a condition in which an adequate amount of oxygen supply is missing in the body, and it could be caused by a variety of diseases, including gastrointestinal disorders. This review is focused on the role of hypoxia in the maintenance of the gut homeostasis and related treatment of gastrointestinal disorders. The effects of hypoxia on the gut microbiome and its role on the intestinal barrier functionality are also covered, together with the potential role of hypoxia in the development of gastrointestinal disorders, including inflammatory bowel disease and irritable bowel syndrome. Finally, we discussed the potential of hypoxia-targeted interventions as a novel therapeutic approach for gastrointestinal disorders. In this review, we highlighted the importance of hypoxia in the maintenance of the gut homeostasis and the potential implications for the treatment of gastrointestinal disorders.

## 1. Introduction

Oxygen is essential for mammals to survive, including humans. It is also a key factor in several biochemical reactions involved in the normal physiology of the body. At the same time, it may be also converted into reactive oxygen species (ROS), which was reported to have negative impact on cells. Hypoxia-inducible factor (HIF) could activate gene transcription and modulate the cellular oxygen levels and metabolic activity (Choudhry and Harris, 2018), playing a role in the oxygen sensing within mammalian cells (Agani and Jiang, 2013), demonstrated by investigations about the erythropoietin regulation. Since its discovery, the number of known HIF and hypoxia-regulated target genes increased, including the ones encoding proteins of several and important biological processes, such as erythropoiesis, angiogenesis, glycolytic pathway, glucose transport, metastasis, and cell survival (Cummins et al., 2008; Colgan and Eltzschig, 2012). As a result, HIF was identified as the most involved factor in the regulation of the cellular responses to oxygen deprivation (Agani and Jiang, 2013). Indeed, the HIF and its related signaling pathways are essential to facilitate the metabolic adaptation to hypoxia-induced stress (Jin et al., 2018; Zhong et al., 2020). Additionally, HIF is involved in many important physiological processes such as cardiovascular generation (Semenza, 2014), tumor progression (Palazon et al., 2017), and pulmonary hypertension (Luo et al., 2019). Over the past decade, the importance of intestinal microbiota in human physiological and metabolic functions has been widely recognized. At the same time, research shows that HIF also plays an important role in regulating intestinal function (Kumar et al., 2020). Specifically, HIF-1α facilitates the host-microbe crosstalk maintaining the gut homeostasis (Fan et al., 2015) as it up-regulates the tight junction proteins, thereby improving the integrity of the epithelial barrier, leading to a resolution of gut inflammation while facilitating the microbial colonization (Kumar et al., 2020). The mucin induced by HIF-1α promotes the colonization of commensal bacteria within the mucus layer, an essential component of the innate immune system, forming a defensive physical barrier for pathogens at the intestinal epithelium level (Kang et al., 2022). Substantial evidence indicated that HIF-1α contributed to the development of disease, and that it can be considered as a promising therapeutic target due to its involvement in the intestinal homeostasis maintenance. This review comprehensively discussed the HIF-1α structure and function, the associated signaling pathways, its role in the disease development, as well as the impact on the intestinal homeostasis.

## 2. Structure and function of HIF

Semenza et al. (1991), Ratcliffe (2007), and Pugh et al. (1991) discovered a transcriptional enhancer able to control the expression levels of erythropoietin, which is in turn regulated by the oxygen concentration as it is induced under hypoxic conditions. The hypoxia-inducible gene expression is regulated by a group of proteins recognized as hypoxia-inducible factors (HIFs). In 1992, Semenza et al. (1991) found that HIF-1 is composed of two proteins (HIF-1α and HIF-1β) that mediates the body adaptation to hypoxic conditions through red blood cell and angiogenesis. There are five major isoforms of HIFs, including HIF1α, HIF2α, and HIF3α which are oxygen-sensitive, and HIF1β and HIF1β2 that showed oxygen-insensitive (Hankinson, 2008; Prabhakar and Semenza, 2012; Choudhry and Harris, 2018). HIF-1α and HIF-2α are stable in hypoxic conditions, they form heterodimers with HIF-1β and thus to activate the gene transcription (Smythies et al., 2019). Interestingly, HIF-1α showed high activation during brief stage of severe hypoxia or anoxia, while HIF-2α was found to be more active in mild or physiological hypoxia, and retain continuous active in 48–72 h of hypoxia (Hu et al., 2022). Thus, it may be possible to state that HIF-1 is responsible for initiating the hypoxic response, while HIF-2α plays a more predominant role in driving the prolonged hypoxic response (Koh et al., 2011; Koh and Powis, 2012; Hu et al., 2022).

**Figure 1:**
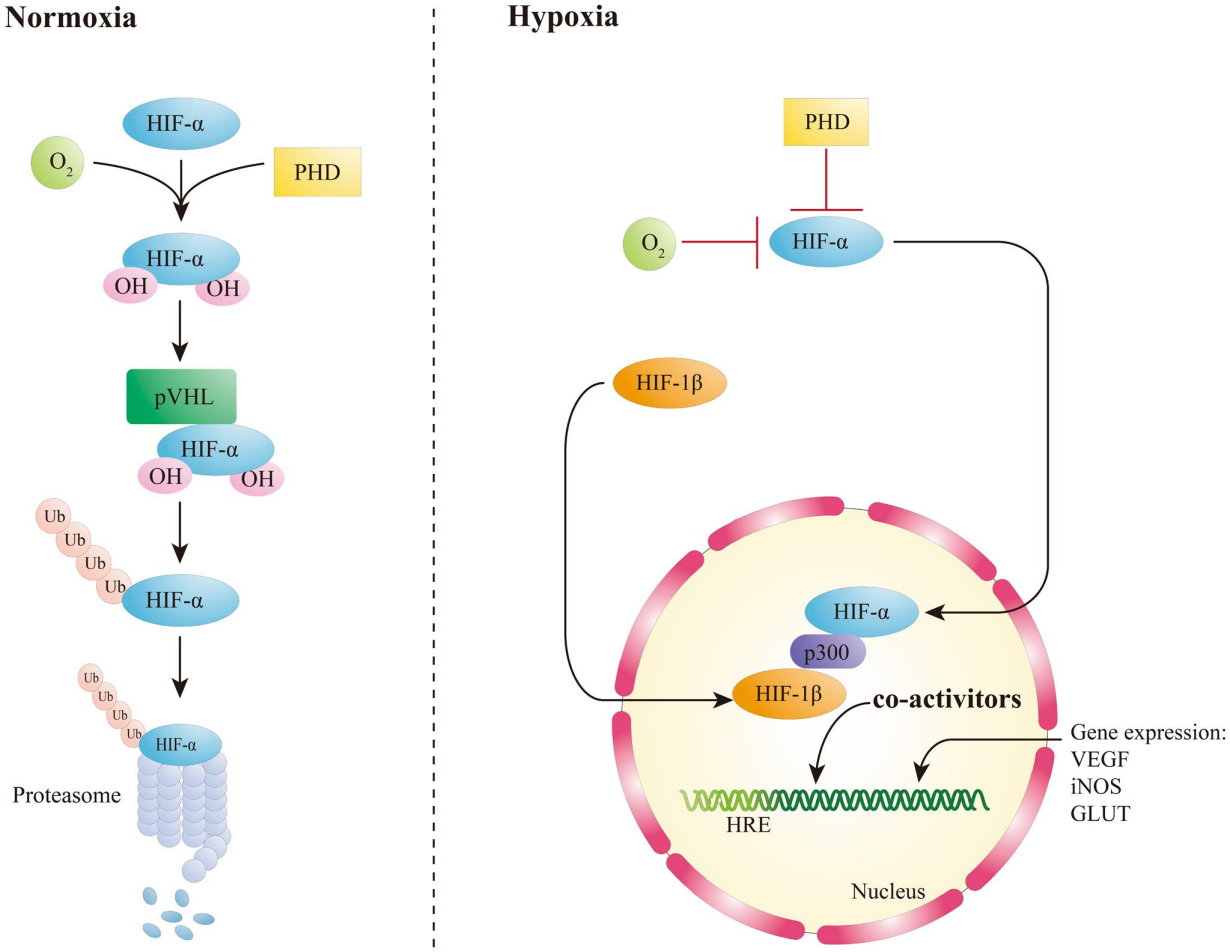
Oxygen-dependent regulation of HIFs.

Being an oxygen-sensitive transcription factor, HIF-1α is correlated with the maintenance of oxygen homeostasis in mammalian cells, and it mediates the adaptive responses to hypoxia (Choudhry and Harris, 2018; Sousa et al., 2019). The protein stability of HIF-1α is mainly modulated by the oxygen-dependent degradation domain (Sun et al., 2017; Zheng et al., 2021). In the presence of oxygen, HIF-1/2α encounters hydroxylation via a specific prolyl hydroxylases (PHDs) at two conserved proline residues (P402/P564 and P405/P531 for human HIF-1α and HIF-2α, respectively) (Hu et al., 2022). After hydroxylation, HIFα is thus identified by von Hippel–Lindau (VHL) to be subsequently degraded through an oxygen-dependent ubiquitin-proteasome pathway (Figure 1) (Sousa et al., 2019). Therefore, under regular conditions, HIF-1α has a very short half-life. The hydroxylation of proline residues in HIFα is critical for VHL binding and relies on PHD, α-ketoglutarate-dependent dioxygenase, and asparagine hydroxylase, that have inhibitory effects on HIF (Choudhry and Harris, 2018). In case of hypoxia, the proteasomal of HIFα is stably expressed and not degraded by the ubiquitin ligase system. HIF-1α is therefore accumulated and it can be translocated to the nucleus, where it binds to aryl carbon receptor nuclear translocator (ARNT) to form the HIF-1α/β heterodimer (Aj et al., 2010; Lee et al., 2019), that binds to p300 to form a transcriptional activation complex targeting the hypoxia response element (HRE) within the DNA that activated the transcription of HIF-1 target genes, such as vascular endothelial growth factor (VEGF), erythropoietin, inducible nitric oxide synthase (iNOS), and glucose transporter (GLUT) (Choudhry and Harris, 2018; Singh et al., 2018). HIF-2α is an heterodimeric transcription factor formed following the dimerization between HIF-2α subunit and its obligate partner subunit nuclear translocator (ARNT) (Wu et al., 2019). Similarly to HIF-1α, HIF-2α is activated in hypoxic conditions through a common signaling pathway (Hu et al., 2022), causing a more relevant response to hypoxia due to its higher affinity for the promoters of genes involved in the hypoxia response (Hu et al., 2022), which results also in an enhanced inducing activity of HIF-2α in some cancers (Wu et al., 2019). The inhibitory PAS domain protein (IPAS), a short splice variant of hypoxia-inducible factor 3 alpha (HIF-3α), has been shown to drive transcriptional activity through its interactions with HIF-1α and HIF-2α in mice, as demonstrated by Tanaka et al. (2009) and Kobayashi et al. (2015). HIF-3α has been traditionally considered as a negative regulator of the hypoxia response pathway (Ravenna et al., 2016). However, recently it was found that long variants of HIF-3α have the capacity to create αβ dimers with an inverse activation ability (Tolonen et al., 2020). HIF-β subunit, also named as the aromatic hydrocarbon ARNT, is not regulated by e oxygen levels. Recent research has revealed that a prolonged hypoxic environment can increase HIF-1β expression in high-risk multiple myeloma cells, with this effect mediated via the expression of nuclear factor kappa-light-chain-enhancer of activated B cells (NF-κB) (Wu et al., 2018).

**Figure 2:**
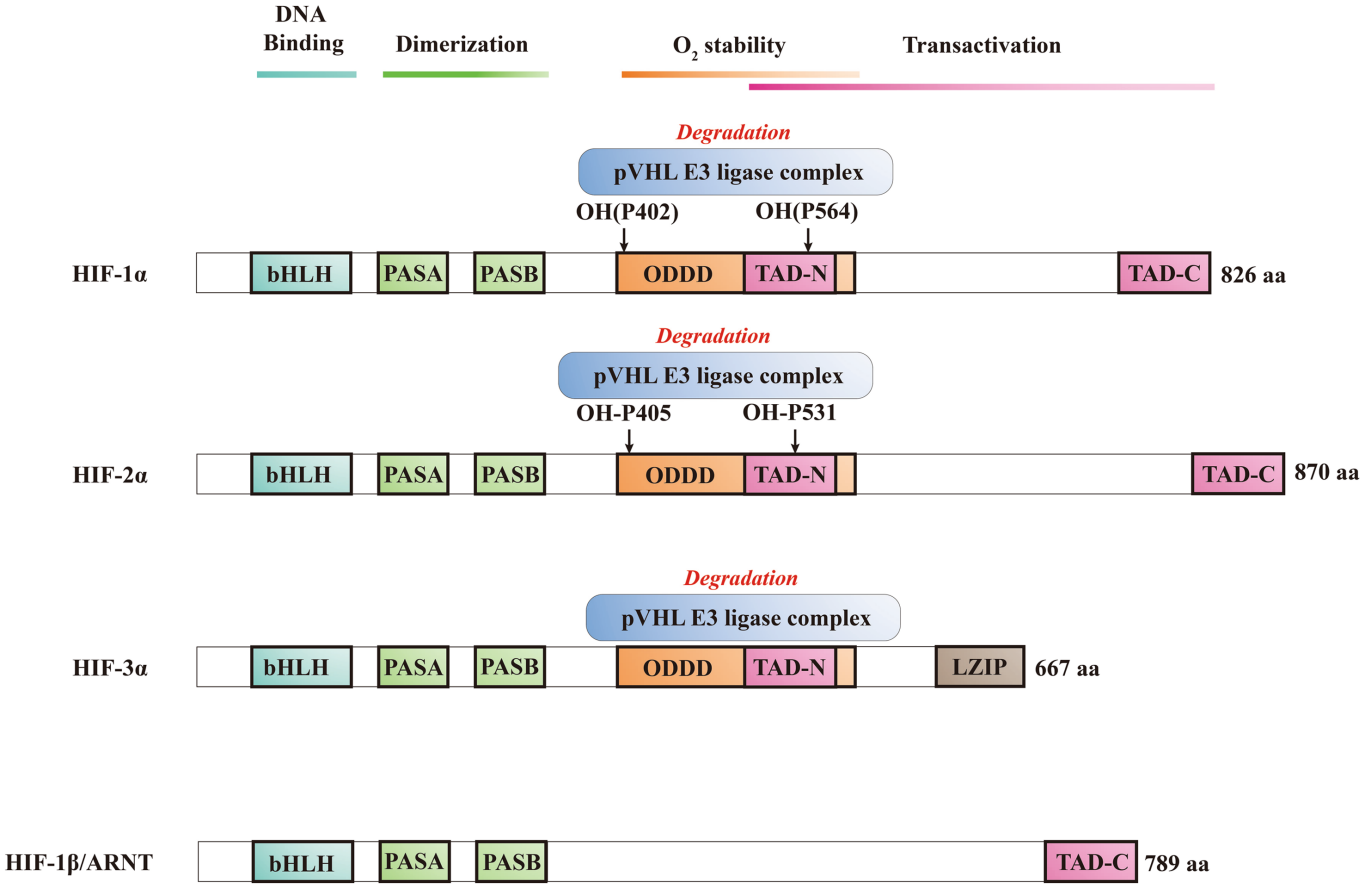
Schematic illustration of the domain structure of HIFs.

**Figure 3:**
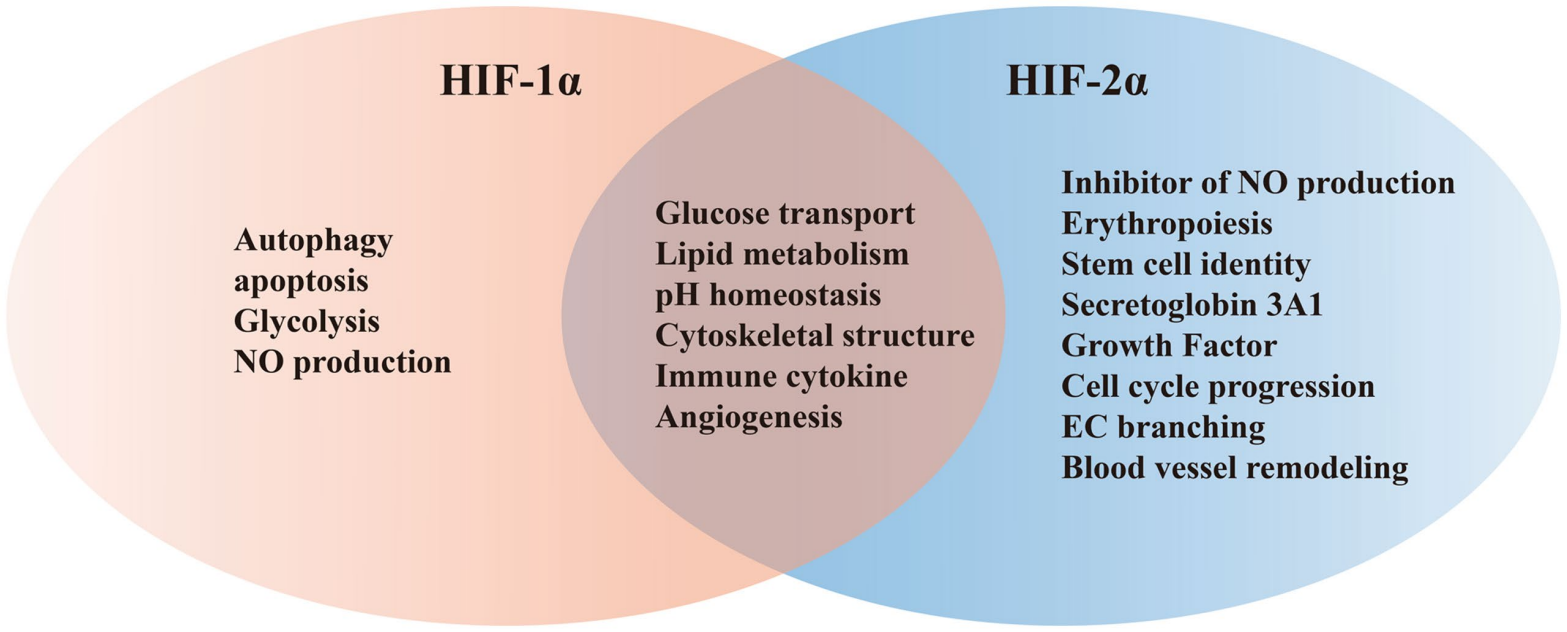
Representative shared and unique target genes regulated by HIF-1α and HIF-2α.

HIFs is classified within the bHLH/PAS protein family, and it is composed of one N-terminal basic-helix–loop–helix (bHLH) domain and two Per-ARNT-Sim (PAS) domains that are crucial for DNA binding and dimerization (Bersten et al., 2013; Yang et al., 2021). Activation of the target gene is facilitated by the N-terminal transactivation domain (NTAD) present in HIFα isoforms, as well as by the C-terminal transactivation domain (CTAD) that is included in both isoforms (Prabhakar and Semenza, 2012). HIF-3α features a unique C-terminal leucine zipper (LZIP) domain that facilitates protein–protein interactions instead of the CTAD. On the other hand, both HIF-1β and HIF-2β lack the ODDD/NTAD and LZIP domains, and while they possess a CTAD, they lack an asparagine residue (Figure 2) (Ravenna et al., 2016). Within the DNA binding and dimerization domains, HIF-1α and HIF-2α shared a high level of homology in both DNA sequence and structure (Smythies et al., 2019). However, substantial evidence suggests that HIF-1α and HIF-2α heterodimers show distinct physiological functions and different roles in the same disease (Rosenberger et al., 2002; Takeda et al., 2010). For example, following the activation of both subtypes in VHL-deficient renal cancer, it was suggested that HIF-2α is oncogenic, whereas HIF-1α has tumor suppressor properties (Shen et al., 2011; Salama et al., 2015). Structurally, HIF-2α and HIF-1α are highly similar, with an overall amino acid (aa) identity of 48%. In particular, their bHLH domains share up to 83% of aa identity, while their PAS regions approximately 70% of aa identity (Hu et al., 2022). Despite a common consensus DNA-binding motif, HIF-1α and HIF-2α bind two distinct but overlapping sites in chromatin with common and unique patterns of downstream gene induction (Koh et al., 2011). Through the examination of the gene induction pattern in renal cell carcinoma 786-O WT-8 cellsstrongly indicates that the upregulation of ADRP, NDRG-1, and VEGF can be attributed to the activity of HIF-2α (Keith et al., 2011). A previous investigation that decreased the expression of specific HIF-α isoforms in Hep3B cells by siRNA revealed a predominant function of HIF-2α in the stimulation of erythropoietin (EPO) production (Shu et al., 2019). Several investigations demonstrated that HIF-1α or HIF-2α could regulate the expression of several genes induced by hypoxia, however, each HIFα isoform showed also distinct targets (Figure 3) (Rr et al., 2005). Several studies suggested that different transcriptional responses mediated by HIF-1α and HIF-2α allow the adaptation to hypoxia (Colgan and Eltzschig, 2012). For example, the ones that coordinate the glycolytic pathway include multiple target genes, and it appears that the HIF-1α subtype is more selective than HIF-2α (Keith et al., 2011). Hif-1α^−/−^ ES cells lost the hypoxic response of the glycolytic genes GLUT-1 and VEGF, suggesting a regulation only by HIF-1α (Keith et al., 2011). In addition, compared with HIF-1α, HIF-2α shows a more prominent effect on the induction of erythropoietin (Shu et al., 2019).

## 3. HIF-1α and signaling pathways

**Figure 4:**
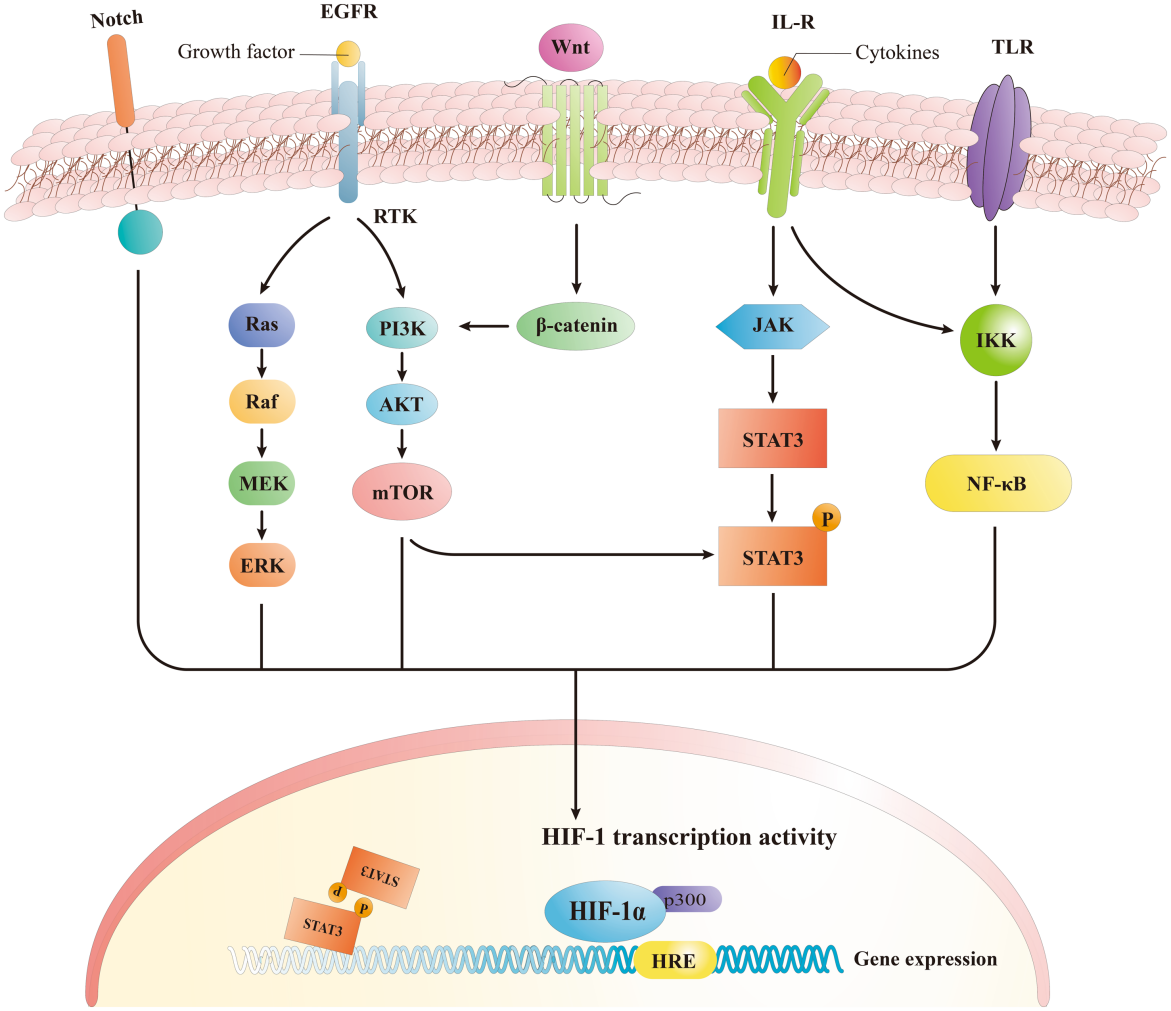
Signaling pathway of HIF-1α. HIF-1α induced expression of downstream genes by activating different signaling pathways.

HIF-1α is located in a wide range of human cells and it interacts with several up-stream and downstream proteins to establish different signaling pathways. HIF-1α mediates the hypoxia signals, leading to a range of compensatory responses to hypoxia, and it plays a significant role in physiological and pathological processes within the body (Xu et al., 2022). The HIF stabilization during the hypoxia is important to upregulate several hundred of downstream target genes, in light of the complexity and importance of HIF signaling (Lee et al., 2019). Most recent research studies indicate that HIF-1α is involved in several signaling pathways, including phosphatidylinositol-3 kinase/protein kinase/mechanistic target of rapamycin (PI3K/Akt/mTOR), extracellular signal-regulated kinase (ERK), Wnt/β-catenin, Notch, and NF-κB (Figure 4) (Malekan et al., 2021; Zhang et al., 2021). These pathways affect several functions including cellular metabolism, regulation of cell proliferation, and control of inflammatory responses (Luo et al., 2022).

Previous research indicated that the phosphatidylinositol-3 kinase/protein kinase B signaling pathway (PI3K/Akt), governs a variety of cellular processes, is able to modulate the HIF-1α expression (Zhang et al., 2018). The downstream localization of HIF-1α is modulated by the mechanistic target of rapamycin complex 1 (mTORC1) pathway, while the PI3K/Akt signaling pathway regulates mTORC1 in an independent way (Malekan et al., 2021). In human mesenchymal stem cells, the exposure to hypoxia stimulated an increase in the levels of both p-Akt and HIF-1α, with p-Akt reaching its maximum earlier than HIF-1α (Zhang et al., 2018). The Akt inhibitor, wortmannin, is also able to inhibit the expression of HIF-1α while mTOR is Akt specific and represent a target of Akt during phosphorylation (Zhang et al., 2018). mTOR functions as an up-stream mediator of HIF-1α activation, and it was recently demonstrated that PI3K/Akt signaling pathway may regulate HIF-1α through mTOR. This regulation may be on a post-transcriptional protein level, altering HIF-1α (Liu et al., 2014). The activation of platelet-derived growth factor (PDGF), transforming growth factor (TGF), tumor necrosis factor-α (TNF- α), and inter-leukin-1β (IL-1β) triggers the HIF-1α regulation through the PI3K/Akt pathway (Oktay et al., 2007; Niu et al., 2008), that lead to an enhanced expression of HIF-1α when activated by receptor tyrosine protein kinase (RTK) (Xie R. et al., 2019). All together these findings suggest that PI3K/Akt/mTOR signaling pathway regulates the HIF-α mRNA levels.

NF-κB is a transcription factor involved in different biological processes including apoptosis, viral replication, tumorigenesis, inflammation, and autoimmune diseases (Barnabei et al., 2021). Endothelial cells (EC) were found able to autocrine TNF-α in case of hypoxia, and to activate the HIF pathway through a NF-κB-dependent process, while producing VEGF that led to the neovascularization (Jin et al., 2019). The silencing of HIF1α and specific glycolytic enzymes can reduce the NF-κB activation and ex-pression of pro-inflammatory genes in endothelial cells when exposed to a disordered flow (Wu et al., 2017). Consequently, endothelial cells when exposed to low and turbulent flow increase the HIF-1α expression and inflammatory signaling by enhancing the NF-κB activation while upregulating the number of glycolytic enzymes (Li et al., 2017). The principal mechanism underlying the canonical NF-κB activation involves a site-specific phosphorylation of IκBα by the multi-subunit IκB kinase (IKK) complex, that leads to its inducible degradation (LaGory and Giaccia, 2016). Furthermore, the activation of the non-canonical NF-κB pathway (i.e., TNFSF14/LIGHT) induces an in-creased expression of HIF, specifically HIF-2α. This is also facilitated by direct interaction of NF-κB subunit p52 and HIF-2α, that initiates multiple site binding on the subunit (Wu et al., 2017). In previous studies, we observed that TNFα, which is a canonical activator of NF-κB, may also be involved in the activation of the HIF pathway in ECs (Yao et al., 2015). Studies have shown that the anti-angiogenic activity of low-density lipoprotein (LDL) is focused on the reduction of HIF-1α and HIF-2α protein levels in ECs, and this is possibly related to the inactivation of NF-κB and down-regulation of HIF-1β (Yao et al., 2015). Inflammatory stimulants and other factors can increase the HIF-1 gene and protein level expressions by modulating NF-κB-dependent signaling (Xu et al., 2022). Other studies demonstrated that the NF-κB pathway can activate the expression of HIF-2α mRNA in osteoarthritis following the increased HIF-2α expression in mouse articular chondrocytes following the treatment with IL-1β, which is a stimulator of the NF-κB pathway (Yang et al., 2010). Additionally, icariin regulated the NF-κB/HIF-2α axis and reduced the inflammation in chondrocytes (Wang et al., 2020), and NF-κB signaling was found to stimulate the expression of HIF-1β (van Uden et al., 2011).

The ERK pathway represent another key pathway triggering the expression of HIF-1α by increasing the HIF-1α protein generation (Wan and Wu, 2016; Luo et al., 2022). It is important to note that the ERK pathway not only regulates the synthesis of HIF-1α but also phosphorylates the coactivator CBP/p300, thus enhancing the formation of the HIF-1α/p300 complex (Malekan et al., 2021). Hyperthermia induces the expression of HIF-1α in lung cancer through the AKT and ERK signaling pathways (Wan and Wu, 2016). In addition, it was demonstrated that photodynamic therapy (PDT) increases the expression of HIF-1α through the ROS-ERK axis, thereby enhancing the resistance to the treatment (Lamberti et al., 2017), indicating a regulation role of ERK signaling on the HIF-1α expression.

Besides the signaling pathways mentioned above, Wnt/β-catenin and Notch pathways are also involved in the modulation of HIF signaling. HIF-1α indeed acts downstream and upstream of the Wnt/β-catenin signaling pathway indicating their mutually regulating functions (Liu et al., 2015; Wu et al., 2015). Specifically, Wnt/β-catenin regulates the function of HIF-1α by initiating the PI3K/Akt signaling pathway (Laukoetter et al., 2008; Lau et al., 2011). The high expression of HIF-1α resulting from hypoxia in cells can activate the Wnt/β-catenin signaling pathway and thus increasing the β-catenin (Wu et al., 2015), while HIF-1α signal also regulates Wnt/β-catenin pathway via calreticulin (Liu et al., 2021). In turn, as an upstream pathway through related signaling pathways, Wnt/β-catenin can indirectly regulate the HIF-1α expression. Recently, it was also demonstrated the role of Notch/HIF-1α signaling in different processes like liver regeneration, angiogenesis, and cancer epithelial-mesenchymal-transition (Li et al., 2020). It was reported that the downstream Janus kinase (JAK)-STAT3 signaling pathway is activated by IL-6, leading to an increase in HIF-α. Notably, this mechanism is consistent with the fact that mTORC1 phosphorylates STAT3, and is involved in the upregulation of HIF-1α mRNA expression (LaGory and Giaccia, 2016).

## 4. HIF and gut homeostasis

In the recent years, it was extensively described that the gut microbiota plays a crucial role for the balance between health status and disease development of host species. The microbiota can be considered as a distinct “human organ” (Shepherd et al., 2018; Marietta et al., 2019). The establishment, selection, and colonization of microbes are governed by a complex molecular network of host-gut microbiota interactions (Kumar et al., 2020). Specifically, several clinical studies showed that hypoxia and inflammation are present in the tissue microenvironment in several inflammatory diseases (Malkov et al., 2021). At a cellular level, the primary control underlying the tissue adaptation to hypoxia is through the HIF signaling pathway (Malkov et al., 2021), and in particular HIF-1α is considered a key regulator of hypoxic injury. In case of IBD, not only the entire mucosa can become even more hypoxic and the expression of HIF-1α and HIF-2α may also be elevated in intestinal surgical specimens (Eltzschig and Carmeliet, 2011). Intestinal epithelial cells (IECs) are thus exposed to the hypoxic environment within the intestinal lumen and represent a major site for host–microbe interactions in order to modulate physiological outcomes (Muenchau et al., 2019; Kumar et al., 2020). The intestinal epithelium not only is re-sponsible for the transfer of nutrients, water, and electrolytes from the lumen to the underlying tissues, but also plays a crucial role in maintaining gut homeostasis by serving as a physical and immunological barrier that prevents the entry of commensal bacteria and potentially harmful microorganisms (König et al., 2016). Altered intestinal barrier function is known to increase susceptibility to enteric infections and disrupt the physiological mechanisms responsible for maintaining tolerance to commensal microorganisms. These changes can ultimately lead to chronic gastrointestinal inflammation and the development of inflammatory bowel diseases, including Crohn’s disease (CD) and ulcerative colitis (UC) (Konjar et al., 2021).

### 4.1. The effect of HIF on the intestinal barrier

**Figure 5:**
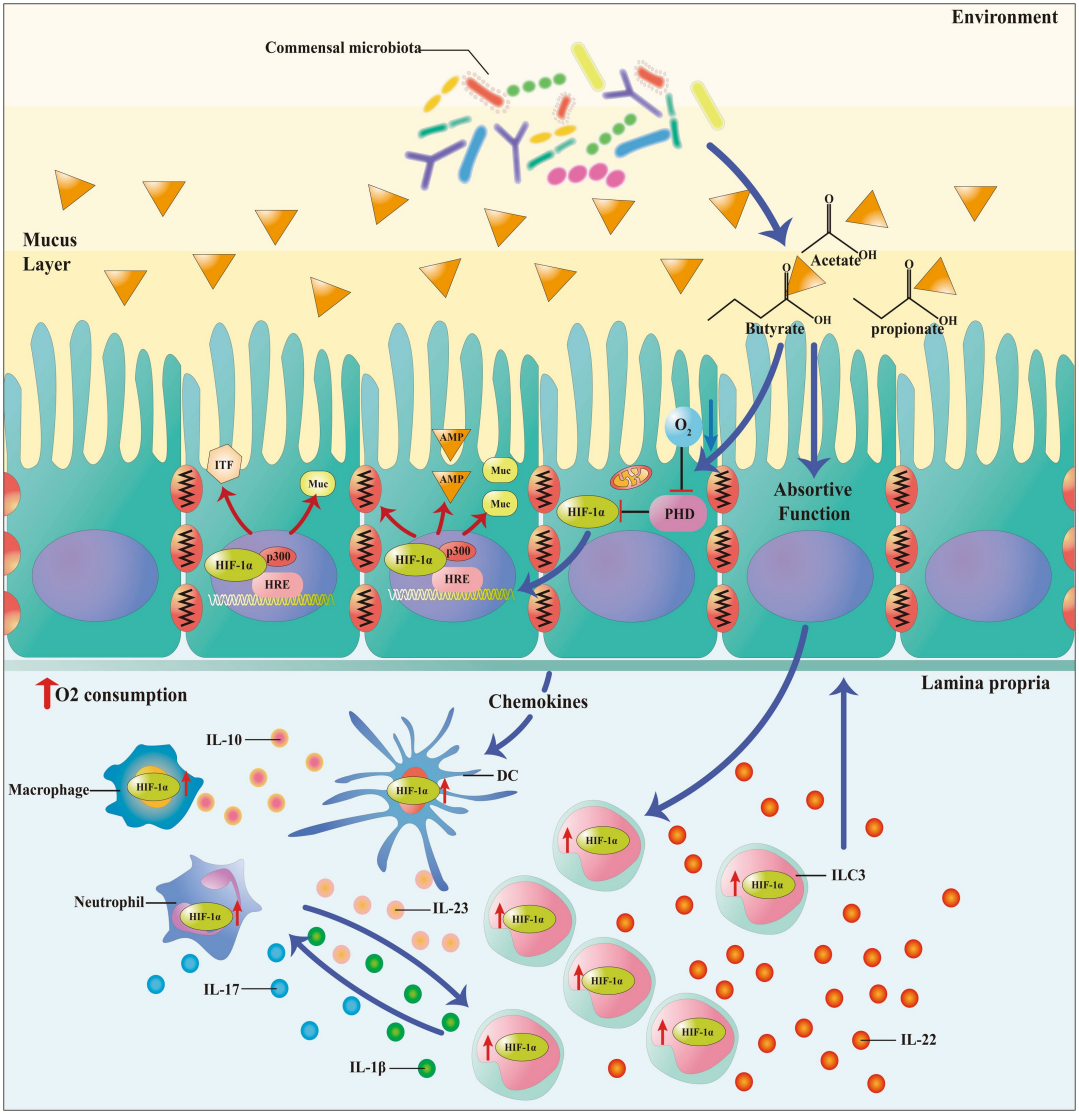
HIF-1α plays an important role in maintaining the intestinal homeostasis as it enhances the epithelial barrier integrity by upregulating the expression of genes, thereby reducing intestinal inflammation, and promoting microbial colonization. IEC and other cellular activation (such as ILC3) release anti-inflammation cytokines and increase the oxygen consumption to stabilize the expression of HIF-1α.

Studies have found that HIF-1α may play a key role in maintaining intestinal homeostasis by regulating the integrity of the intestinal epithelial barrier while cultivating a suitable ecological niche (Kumar et al., 2020). Additionally, HIF-1α maintains the intestinal epithelial integrity by upregulating genes involved in the maintenance of the intestinal barrier integrity, such as muc2, ITF, cldn1 as well as other tight junction proteins (Figure 5) (Kumar et al., 2020). Given that HIF-1α plays a direct role in preserving the intestinal epithelial integrity by promoting barrier functionality (Muenchau et al., 2019), the increased integrity of the gut barrier may have immunosuppressive effects, as it seals paracellular pathways while inhibiting the immune cells activation (Chelakkot et al., 2018). Both HIF-1α and HIF-2α are expressed in the intestinal epithelial cells of ulcerative colitis and Crohn’s disease patients and in mouse models of colitis (Xue et al., 2013). In a model of radiation-induced intestinal toxicity, HIF-2α restores epithelial integrity and reduces apoptosis by inducing angiogenic gene expression (Olcina and Giaccia, 2016). Following intestinal injury, HIF-2α directly regulates chemokine/cytokine networks to recruit neutrophils and multiple pro-inflammatory mediators to eliminate noxious stimuli and restore the mucosal barrier (Singhal and Shah, 2020). The differentiation of regulatory T (Treg) (Lei et al., 2015) cells plays a crucial role in the establishment and proliferation of the human gut microbiome (Luu et al., 2017). In particular, under cellular hypoxic conditions, HIF-1α promotes the differentiation of naive CD4 cells into regulatory T cells by inducing the transcription of FoxP3., and the anti-inflammatory cytokine IL-10 is produced, to downregulate the immune response, thereby reducing the colonic inflammation while promoting the immune tolerance (Luu et al., 2017). Extracellular ATP is subject to enzymatic hydrolysis by nucleoside triphosphate dephosphorylase (NTPDase or CD39) to generate AMP, which is in turn converted into adenosine by Ecto-5′- Nucleotidase (5-NT or CD73) (Allard et al., 2017). Adenosine exerts immune-modulatory effects and promotes the enhancement of the epithelial barrier by activating A2B adenosine receptors, that then trigger the phosphorylation of vasodilator-stimulated phosphoprotein (VASP) (Aherne et al., 2015). Elevated expression levels of HIF-1α were evidenced in patients with Crohn’s disease and ulcerative colitis, indicating its protective role in mitigating inflammatory bowel disorders by improving the epithelial barrier functionality (Shah, 2016).

### 4.2. Interaction of NF-κB and HIF-1α in the intestine

The maintenance of gut homeostasis depends on an intricate functional crosstalk between HIF-1α and NF-κB signaling pathways. Specifically, HIF-1α inhibits TAK1, which in turn prevents the downstream activation of IKK, ultimately leading to downregulation of NF-κB activity and decreased inflammation (Liu et al., 2017). The transcription factor NF-κB is involved in the expression of numerous genes involved in immune response, serving as a key mediator of inflammatory responses. Specifically, NF-κB induces the expression of numerous pro-inflammatory genes, which encoding cytokines and chemokines, and also is involved in the regulation of inflammasomes (Cutolo et al., 2022). HIF-1α pathway indeed strongly affects the epithelial and immune system function and development during inflammation by activating an adaptive response in these cells (Taylor et al., 2016). The pro-inflammatory function of NF-κB has been widely investigated in macrophages, which are innate immune cells located across several tissues with defensive functions against infectious agents. Notably, the constitutive activation of NF-κB was detected in the inflamed colonic tissue of individuals diagnosed with IBD (Liu et al., 2017). NF-κB serves as a pivotal transcription factor in M1 macrophages and plays a crucial role in inducing a multitude of inflammatory genes, such as those encoding TNF-α, IL-1β, IL-6, IL-12p40, and cyclooxygenase-2 (Wang et al., 2014). A recent study employing dextran sulfate sodium (DSS)-induced colitis mouse model reported that the absence of HIF-1α in dendritic cells leads to intestinal inflammation through the upregulation IL-6 and IL-23 (Flück et al., 2016). Additionally, HIF-1α can activate NF-κB under hypoxic conditions and enhance the expression of TNF-α (Gerri et al., 2017; Gunton, 2020). Furthermore, HIF-1α-induced NF-κB pathway increases TLRs expression while inducing an inflammatory cascade leading to ECs injury (Groschwitz and Hogan, 2009; Gerri et al., 2017). In turn, downregulation of HIF-1α expression can effectively reduce the inflammation and oxidative stress-induced damage during EC stress in hyperglycemia-induced mice (Liu et al., 2018; Xie Y. et al., 2019). The stabilization of HIF-1α conferred protective effects by attenuating the NF-κB signaling pathway, and thus reducing the cellular inflammation (Bandarra et al., 2015). Given the potential therapeutic properties of HIF for inflammatory disorders, further investigations into the role of inflammatory cytokines in the HIF pathway may provide novel therapeutic insights into the management of inflammatory diseases.

### 4.3. Hypoxia-mediated effects of HIF-1α and SCFAs

Microbiota-derived short-chain fatty acids (SCFAs) have gained a growing attention in recent years. The microbial supply for SCFAs, especially butyrate, is a recognized contributor to the gut homeostasis and disease resistance, while is used as a favorable energy source for enterocytes of the colon (Wang et al., 2021). The mucus layer, secreted by goblet cells, functions as a protective barrier against both endogenous and exogenous irritants, as well as microbial adhesion and invasion (Kang et al., 2022). As a vital element of the innate defense system, the mucosal barrier not only contributes significantly to mucosal repair but also safeguards the mucosal epithelium from a range of injuries within the gastrointestinal tract (Guo et al., 2023). SCFAs increase oxygen consumption by intestinal epithelial cells, reduce their availability in the intestine and lead to hypoxia (Bersten et al., 2013; Pral et al., 2021). The decrease in oxygen utilization stabilizes the expression of HIF-1α and translocates it to the nucleus, causing the transcriptional expression of multiple genes (Choudhry and Harris, 2018). Stable expression of HIF-1α upregulates the expression of related genes such as MUC2, MUC3, and intestinal trefoil factor (Guo et al., 2023). Furthermore, related studies have shown that mice lacking HIF-1α exhibit less organized and diffusible mucin granules, suggesting that HIF-1α is necessary for mucin processing and maintenance of mucosal integrity (Kumar et al., 2020). Fachi et al., demonstrated in a mouse model that butyrate increases the expression of claudin in a HIF-1α-dependent manner, leading to improved barrier integrity and reduced inflammation by inhibiting microbial translocation (Fachi et al., 2019; Muenchau et al., 2019), and HIF-1α was found to be required for butyrate protection of the intestinal epithelium in a mouse model of *Clostridium difficile* infection (CDI) (Fachi et al., 2019). Butyrate inhibits PHDs leading to the stabilization of HIF-1α, which in turn upregulates the expression of genes involved in intestinal barrier function, including muc2, ITF, cldn1, and other tight junction proteins (Kumar et al., 2020). Kelly et al. (2015) demonstrated that the treatment of Caco-2 cells with butyrate resulted in a decreased barrier permeability. Studies demonstrated that the knockdown of HIF-1β using specific shRNA in T84 and Caco-2 cells resulted in a decrease in the expression of claudin-1 at both mRNA and protein levels along with defects in barrier function and abnormal morphology of tight junctions (Muenchau et al., 2019). The researchers found that the gut barrier was weakened, HIF-1α was activated and the HIF-1αΔIEC phenotype was reversed during 2,4,6-trinitrobenzenesulfonic acid (TNBS) colitis in a mouse model (Karhausen et al., 2004; Holmquist-Mengelbier et al., 2006). Hirota et al., reported an alleviation of intestinal injury and inflammation induced by *C. difficile* in mice expressing HIF-1α in IECs compared to Hif1α-deficient mice (Hu et al., 2003; Hirota et al., 2010). Taken together, these findings suggest that SCFAs increase the oxygen consumption of intestinal epithelial cells, stabilize the expression of HIF-1α, and upregulate the gene expression of tight junction proteins and intestinal barrier function, maintaining mucosal and epithelial barrier integrity, thereby reducing intestinal inflammation and promoting Microbial colonization.

### 4.4. HIF-1α and SCFA-mediated regulation of ILC3s

Innate lymphoid cells (ILCs) play a crucial role in regulating mucosal immunity, inflammation, and tissue homeostasis.” ILCs includes cytotoxic cells (NK cells) and “helper-like” ILCs, that are primarily tissue-resident cells and play a vital role in tissue homeostasis, inflammation, and mucosal immunity. These cells act rapidly in the immune response, responding to signals or inducer cytokines that are expressed by tis-sue-resident cells (Vivier et al., 2018). ILC3s are highly prevalent in mucosal tissues and play a crucial role in the innate immune response against extracellular bacteria and in the containment of intestinal commensals (Rankin et al., 2016). SCFAs regulates the number of ILC3s in peripheral tissues and attenuates rodent infection in mouse gut by promoting cytokine signaling and activating the mammalian target of rapamycin (mTOR) pathway (Sepahi et al., 2021). ILC3s produce the predominant homeostatic cytokine, IL-22, which is important for the maintenance of the intestinal homeostasis and proliferation of intestinal stem cells (Figure 5). Studies using antibodies against IL-22 and IL-22 knockout mice have demonstrated the role of this cytokine in alteration of the gut microbiota by stimulating the production of antimicrobial peptides (AMPs) such as the regenerating protein RegIIIγ (Lo et al., 2019). Additionally, activation of hypoxia/HIF-1α signaling was shown to enhance murine resistance to *C. difficile* infection. This was demonstrated by an improvement in clinical scores together with a reduction in intestinal bacterial translocation in infected wildtype (WT) mice com-pared to mice with a conditional RORyt-specific HIF-1α knockout (HIF-1αfl/flRORc-Cre) (Fachi et al., 2021). Several studies have also demonstrated that butyrate has anti-inflammatory effects on M1 macrophages stimulated with LPS, as it is capable of reducing the production of pro-inflammatory mediators such as NO and IL-6 (Chang et al., 2014). M2 macrophages are involved in the resolution of inflammation and tissue repair by producing anti-inflammatory cytokines such as IL-10 and IL-13, as well as growth factors and extracellular matrix proteins (Cekic and Linden, 2016; Liu et al., 2017). Commensal microbe-derived butyrate, as a novel effector molecule, can ameliorate DSS-induced colitis in mice by reducing the activation of M1 macrophages and promoting the differentiation of regulatory T cells. DCs treated with butyrate show less ability to stimulate T cells with a reduction in the production of the pro-inflammatory cytokines IL-12p40 and IFN-γ, while instead releasing greater amounts of the an-ti-inflammatory cytokine IL-10 (Liu et al., 2012).

### 4.5. HIF-1α and intestinal homeostasis mediate alcoholic liver disease

Previous research showed that alcoholic liver disease (ALD) is associated with gut dysbiosis and release of endotoxins (Shao et al., 2018). Shao et al. demonstrated that HIF-1α plays a critical role in regulating the expression of genes involved in maintaining intestinal homeostasis, including those involved in hepatic lipogenesis, maintenance of intestinal barrier function, antimicrobial defense, and the normal microbiome (Zhao et al., 2010). Goblet cells, a specialized cell type within the intestinal epithelium, are responsible for producing protective trefoil factors and mucins, which are heavily core glycosylated and can be found within the cell membrane or secreted into the lumen where they can form the mucus layer (Zhao et al., 2010; Shao et al., 2018), which is the first barrier encountered by bacteria and that needs to be penetrated in order to reach the epithelial cells (Johansson et al., 2008). In IEhif1α^−/−^ mice subjected to alcohol exposure, there is a decrease in intestinal trefoil factor (ITF), claudin-1, and p-glycoprotein, leading to a compromised gut barrier functionality. This leads to an increased concentration of lipopolysaccharide in the serum and *E. coli* protein in the liver (Shao et al., 2018). Long-term and excessive alcohol consumption can lead to intestinal dysbiosis, which leads to increased intestinal permeability and translocation of LPS into the blood. After LPS binds to TLR4 on hepatocytes (including hepatocytes and Kupffer cells), it triggers an inflammatory response and Leads to hepatic steatosis (fat buildup) and inflammation (Shao et al., 2018). Activation of HIF-1α can regulate the gut bacterial homeostasis by increasing the production of anti-microbial peptides. Additionally, HIF-1α stabilization leads to upregulation of P-glycoprotein and tight junction proteins, which help to maintain barrier functions (Chen et al., 2015). Therefore, ALD can be prevented/treated by developing dietary methods and drugs that specifically activate the intestinal HIF-1α.

## 5. Future perspectives

HIF-1α represents a crucial transcription factor produced under hypoxic conditions, and it plays a pivotal role in the regulation of several cellular processes, including angiogenesis, glucose metabolism, apoptosis, and autophagy. Moreover, HIF-1α is involved in the regulation of multiple signaling pathways, being essential during the body growth and development, as well as in several physiological and pathological processes. Diverse HIF isoforms are responsible for physiological and pathological processes, and HIF-1α may be involved in the development of diseases through the regulation of multiple target genes. Comprehensive investigations on HIF-1α better elucidated its regulatory roles in angiogenesis, glucose metabolism, apoptosis, autophagy, and several signaling pathways. It is well to point out that further advancements in HIF-1α-based therapeutic strategies and the related research will gain more attention, and it may lead to the development of potent HIF-1α inhibitors to use for clinical applications, allowing new discoveries and achievements in terms of disease prevention and treatment.

Significant differences in baseline oxygen tension between gastrointestinal mucosal tissues play unique roles in intestinal homeostasis and inflammation. With in-depth research, more and more evidence shows that the intestinal mucosal barrier, as an important component of intestinal immunity, not only serves as a medium for the absorption and exchange of substances between organisms and the environment, but also prevents external antigens from entering the body. The complete composition and function of the intestinal mucosal barrier function is critical for maintaining immune homeostasis. Once the intestinal barrier function is damaged under the action of multiple factors, immune homeostasis will be disrupted and inflammatory responses will be triggered. Hypoxia regulates the expression of hundreds of genes through HIF transcription factors, such as enhancing tight junctions and reducing intestinal permeability, as well as increasing mucus and AMP to protect mucosal integrity. HIF has been extensively studied in the areas of modulating intestinal tissue barrier function, metabolism, and inflammatory and immune responses. Many studies have highlighted the therapeutic potential of targeting hypoxic signaling pathways in intestinal diseases. Therefore, hypoxia and activation of the HIF pathway may be considered as putative therapeutic targets for the treatment of certain inflammatory and/or infectious diseases, particularly those affecting the gut such as CDI and IBD. Therefore, further studying the interaction between HIF and intestinal microorganisms may be a new strategy for preventing and treating different diseases in the future.

## Author contributions

WL: Data curation, Investigation, Software, Writing – original draft. XF: Data curation, Software, Writing – original draft. BJ: Writing – original draft, Writing – review & editing. DW: Software, Writing – review & editing. HW: Data curation, Writing – review & editing. ZL: Conceptualization, Writing – review & editing. BL: Funding acquisition, Writing – review & editing.
